# Investigating Best Practices for Ecological Momentary Assessment: Nationwide Factorial Experiment

**DOI:** 10.2196/50275

**Published:** 2024-08-12

**Authors:** Michael S Businelle, Emily T Hébert, Dingjing Shi, Lizbeth Benson, Krista M Kezbers, Sarah Tonkin, Megan E Piper, Tianchen Qian

**Affiliations:** 1 TSET Health Promotion Research Center Stephenson Cancer Center University of Oklahoma Health Sciences Center Oklahoma City, OK United States; 2 Department of Family and Preventive Medicine University of Oklahoma Health Sciences Center Oklahoma City, OK United States; 3 School of Public Health University of Texas Health Science Center at Houston Houtson, TX United States; 4 Department of Psychology University of Oklahoma Norman, OK United States; 5 Institute for Social Research University of Michigan Ann Arbor, MI United States; 6 Center for Tobacco Research and Intervention Department of Medicine University of Wisconsin, Madison Madison, WI United States; 7 Department of Statistics University of California, Irvine Irvine, CA United States

**Keywords:** ecological momentary assessment, mobile health, smartphone, compliance, ambulatory assessment, adherence, experience sampling, mobile phone, mHealth, real-time data, behavior, dynamic behavioral processes, self-report, factorial design

## Abstract

**Background:**

Ecological momentary assessment (EMA) is a measurement methodology that involves the repeated collection of real-time data on participants’ behavior and experience in their natural environment. While EMA allows researchers to gain valuable insights into dynamic behavioral processes, the need for frequent self-reporting can be burdensome and disruptive. Compliance with EMA protocols is important for accurate, unbiased sampling; yet, there is no “gold standard” for EMA study design to promote compliance.

**Objective:**

The purpose of this study was to use a factorial design to identify optimal study design factors, or combinations of factors, for achieving the highest completion rates for smartphone-based EMAs.

**Methods:**

Participants recruited from across the United States were randomized to 1 of 2 levels on each of 5 design factors in a 2×2×2×2×2 design (32 conditions): factor 1—number of questions per EMA survey (15 vs 25); factor 2—number of EMAs per day (2 vs 4); factor 3—EMA prompting schedule (random vs fixed times); factor 4—payment type (US $1 paid per EMA vs payment based on the percentage of EMAs completed); and factor 5—EMA response scale type (ie, slider-type response scale vs Likert-type response scale; this is the only within-person factor; each participant was randomized to complete slider- or Likert-type questions for the first 14 days or second 14 days of the study period). All participants were asked to complete prompted EMAs for 28 days. The effect of each factor on EMA completion was examined, as well as the effects of factor interactions on EMA completion. Finally, relations between demographic and socioenvironmental factors and EMA completion were examined.

**Results:**

Participants (N=411) were aged 48.4 (SD 12.1) years; 75.7% (311/411) were female, 72.5% (298/411) were White, 18.0% (74/411) were Black or African American, 2.7% (11/411) were Asian, 1.5% (6/411) were American Indian or Alaska Native, 5.4% (22/411) belonged to more than one race, and 9.6% (38/396) were Hispanic/Latino. On average, participants completed 83.8% (28,948/34,552) of scheduled EMAs, and 96.6% (397/411) of participants completed the follow-up survey. Results indicated that there were no significant main effects of the design factors on compliance and no significant interactions. Analyses also indicated that older adults, those without a history of substance use problems, and those without current depression tended to complete more EMAs than their counterparts. No other demographic or socioenvironmental factors were related to EMA completion rates. Finally, the app was well liked (ie, system usability scale score=82.7), and there was a statistically significant positive association between liking the app and EMA compliance.

**Conclusions:**

Study results have broad implications for developing best practices guidelines for future studies that use EMA methodologies.

**Trial Registration:**

ClinicalTrials.gov number NCT05194228; https://clinicaltrials.gov/study/NCT05194228

## Introduction

Ecological momentary assessment (EMA) is a measurement methodology that involves the repeated collection of real-time data on participants’ behavior and experience in their natural environment [1]. EMA has been used in behavioral science for years [2], including research examining the environmental and psychological antecedents of cigarette smoking, substance use disorders, anxiety, eating, and sleep [3-6]. EMA sampling strategies are typically time-based or event-based [7]. Time-based sampling often involves prompting EMAs on an interval or random schedule; for example, a daily diary prompted every day at the same time, or at a randomized time within a specific window (eg, every 3-4 hours) each day [1]. In contrast, event-based sampling is typically initiated by a participant when specific events occur. For example, a participant may be asked to initiate an assessment every time they smoke a cigarette [1].

The EMA methodology has several advantages over more traditional lab or clinic-based sampling strategies [1], including (1) minimization of recall bias due to frequent and real-time assessments, (2) more granular examination of the contextual associations between behaviors and psychological and environmental variables, and (3) examination of behavioral trends over time and across situations and settings. While EMA allows researchers to gain valuable insights into dynamic behavioral processes, the need for frequent self-reporting can be burdensome and disruptive to participants [1]. As a result, missing data due to poor compliance with EMA protocols can undermine the validity of the data. Although there are no “gold standards” for EMA protocols, the development of balanced procedures to maximize data coverage and minimize participant burden is important for accurate, unbiased sampling [7,8].

Few studies have experimentally examined the impact of EMA design features on participant compliance. Stone et al [9] randomized 91 patients with chronic pain to 1 of 4 EMA monitoring intensities (ie, 0, 3, 6, or 12 EMAs per day) for 14 days. Results indicated that compliance with the EMA protocol was not related to sampling density. Eisele et al [10] assigned a sample of 152 college students to complete 30 or 60 item EMAs 3, 6, or 9 times per day. Results showed that compliance was lower for those assigned to complete EMAs with more items, but sampling frequency was unrelated to compliance. Finally, Hasselhorn et al [11] randomized college students into 2 separate 14-day studies to examine relations between the number of EMAs prompted per day and EMA compliance (ie, 3 or 9 EMAs per day, N=313; study 1) and the number of EMA items per assessment and EMA compliance (ie, 33- or 82-item EMAs 3 times per day, N=282; study 2). Findings indicated that neither the number of EMAs per day nor the number of items per EMA were related to EMA compliance.

A number of meta-analyses and systematic reviews have also been conducted to examine how EMA design features may affect compliance. In a recent meta-analysis of compliance with EMA protocols among people who use substances (N=126 studies), there was no evidence that compliance rates were associated with prompt frequency, length of assessment period, or type of device used to prompt EMAs [8]. Another study using a pooled dataset of 10 EMA studies found compliance declined over time, women were more compliant than men, older adults were more compliant than younger adults, and compliance varied significantly depending on the time of day [12]. Ottenstein and Werner’s recent review of 488 EMA studies [13] showed longer study periods and higher total numbers of EMAs were related to lower overall EMA compliance. In a meta-analysis of EMA studies in mental health research, greater compliance was associated with female sex, higher incentives for completing EMAs, having EMA prompts at fixed times, and fewer EMAs per day; however, study duration and average age of the sample were not related to EMA compliance [14]. One of the most comprehensive meta-analyses of EMA studies to date (ie, 105 trials were included) indicated that the median study duration was 7 days, a median of 5 EMAs were prompted per day, EMAs included a median of 8-10 questions, and 81.9% of all EMAs were completed [15]. Findings also indicated that studies that had 3 or fewer daily prompts had greater EMA completion than studies with greater than 3 daily prompts, and studies with less than 27 EMA items had higher completion rates than EMAs with 27 or more items. Finally, effect estimates for predictors of compliance varied widely across studies [15]. While the existing literature can offer some guidance regarding best practices for EMA study design, varied reporting across studies with respect to the definition of compliance, study design elements, and study populations make it difficult to clearly derive best practice guidelines for EMA design. To identify optimal EMA design features, it is necessary to use a randomized controlled trial to support the strongest level of inference.

This study used a factorial design to identify optimal factors, or combinations of factors, for achieving the best compliance rates for smartphone-based EMAs. A factorial design is ideal for exploring these research questions because it is statistically more efficient, as this type of design needs fewer participants to answer questions about each experimental factor of interest and allows researchers to examine interaction effects. The included study factors were specifically selected to inform future studies that aim to use EMA data to tailor just-in-time adaptive interventions (JITAIs). Our hypotheses were that fewer EMA items (ie, 15 vs 25), greater EMA frequency (ie, 4 vs 2 per day), paying participants based upon the percentage of EMAs completed (versus US $1 per EMA completed), fixed EMA prompt type (versus random prompts), and slider-type response scale (vs Likert-type scales) would result in greater EMA completion. Finally, we examined the relation between participant demographics (eg, age and sex) and other variables (eg, time of day, day of week) and EMA completion across the 28-day study period.

## Methods

### Procedures

**Figure 1:**
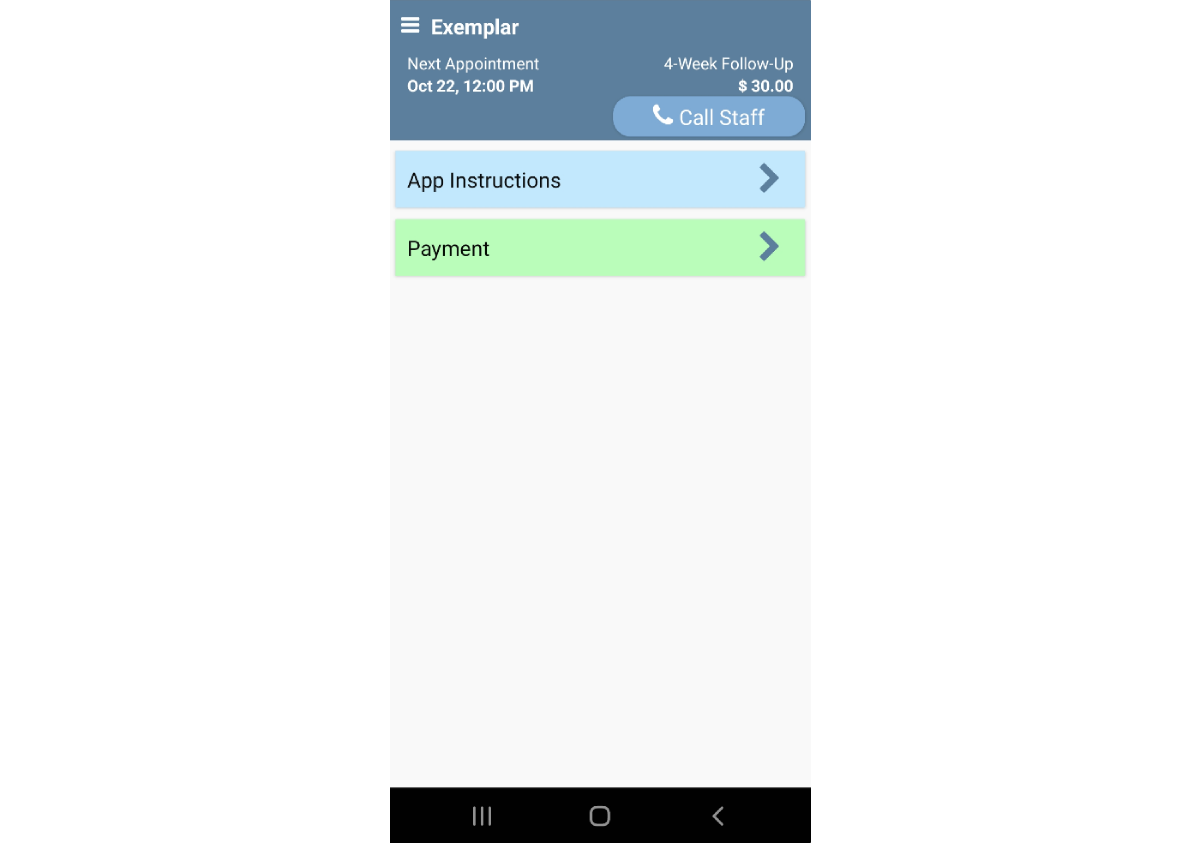
Exemplar home screen.

**Figure 2:**
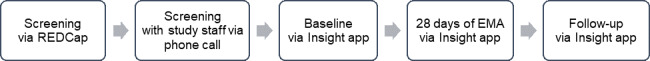
Participant flow.

Participants (N=411) were recruited through nationwide Facebook advertisements from November 30, 2021, through September 27, 2022. One example advertisement read: “We’re looking for Android users to complete brief daily surveys on their smartphones. Qualified participants will be compensated up to $152 over 4 weeks. NO in person visits required.” Those interested in participating clicked a link to a secure, REDCap (Research Electronic Data Capture; Vanderbilt University)-based survey to determine their initial eligibility for the study. Upon screening for the study, all participants were scheduled for a 20- to 30-minute screening and enrollment phone call. During the phone screening, all potential participants completed informed consent electronically via REDCap. Individuals were included in the study if they (1) were ≥18 years of age, (2) demonstrated >6th grade English literacy level, (3) possessed an active Android smartphone with a data plan and an operating system (version 6.0) or higher that was compatible with our Insight smartphone app, (4) agreed to install the Insight app onto their personal phone, (5) texted the study team a picture of their identification, including address (to reduce the likelihood of fraudulent attempts to enroll in the study), and (6) agreed to complete EMAs prompted by and completed through the study smartphone app. Finally, participants were required to identify 14 continuous waking hours each day in which the app could prompt EMAs. Participants were then randomized into 1 of 32 groups, downloaded the Insight app onto their personal smartphone, and were given a unique code to initialize the app. Participants were briefly trained to use the intuitive Insight app for data collection purposes. Participants were instructed to click the “Call Staff” button to speak with study research staff in the event of difficulty with the phone app, and to click the “Payment” button to access an up-to-the-moment accounting of credit earned for all EMAs completed (see Figure 1). All participants completed sample EMA items during the screening phone call to ensure that they understood how to use the app to answer study questions. Participants were given 1 week to complete the baseline assessment in the Insight app. Upon completion of the baseline assessment, participants began the 28-day EMA period. After the 28-day EMA period, participants gained access to a follow-up assessment that was embedded in the Insight app. See Figure 2 for the participant flow through the study. The study smartphone app automatically encrypted and uploaded survey data to our server multiple times per day.

All participants were prompted to complete their assigned EMA battery for the entire 28-day EMA period. The study app automatically prompted brief (ie, approximately 1-3 minutes) EMAs during each individual’s normal waking hours (ie, daily wake and sleep times were collected after participants were screened into the study).

### Study Design

This study was designed to examine the impact of 5 EMA design factors that are relevant and tuned for studies that may use EMA data to inform JITAIs: (1) number of EMA items (15 vs 25 items), (2) EMA frequency (2 vs 4 EMAs per day), (3) payment type (US $1 per completed EMA versus payment based upon the percentage of EMAs completed), (4) EMA prompt type (EMAs prompted at random versus fixed times of the day, assessment times are presented in Table 1), and (5) EMA response scale type (slider-type response scale vs Likert-type response scale). All design factors were specified at the between-person level (ie, each person received 1 level or the other for the entire study period), except for the EMA response scale factor. The EMA response scale factor was specified at the within-person level such that each participant received 1 level (ie, slider scale or Likert scale) for the first 14 days and subsequently received the other level of the factor for the last 14 days of the study. The order of EMA response scale types was counterbalanced across participants, such that approximately half of participants received the slider-type scale for days 1-14 and the Likert-type scale for days 15-28, and the other half of participants received the scale types in the opposite order. The 32 experimental conditions are described in Multimedia Appendix 1.

**Table 1:** Random and fixed ecological momentary assessment (EMA) prompt times.

EMA order	Fixed		Random	
	2 per day	4 per day	2 per day	4 per day
First EMA	30 minutes after waking	30 minutes after waking	First half of waking hours	First quarter of waking hours
Second EMA	—^a^	300 minutes after waking	—	Second quarter of waking hours
Third EMA	—	570 minutes after waking	—	Third quarter of waking hours
Fourth EMA	60 minutes before bed	60 minutes before bed	Second half of waking hours	Fourth quarter of waking hours

^a^Not applicable.

### Study Assessments and Measures

#### Screening Assessment (via REDCap)

Upon viewing the study Facebook advertisements, potential participants self-initiated the initial screening using the secure web-based REDCap platform [16]. Those who met the eligibility criteria were scheduled for a 20- to 30-minute call to complete the informed consent procedure, additional screening items, and confirm the compatibility of their personal smartphone with the Insight app.

#### Baseline and Follow-Up Assessments

The baseline and follow-up assessments were completed via the Insight app. Participants answered questions about their ethnicity (Hispanic or Latino or not), race (more than one race, American Indian, Asian, Black, Native Hawaiian or other Pacific Islander, or White; dichotomized as White versus minoritized race in the current analyses), biological sex, employment status (dichotomized as employed at least part-time or not employed), years of education (continuous), and income (continuous). Other baseline measures included the 5-item Overall Anxiety Severity and Impairment Scale (a measure of current anxiety [17]), the Overall Depression Severity and Impairment Scale (a measure of current depression [18]), and a single item that asked, “Has your use of any of the following substances caused you significant problems?” (check all that apply: alcohol, cannabis, cocaine, K2, opiates, sedatives or hypnotics or anxiolytics, other substances, and none of the above). A single question was used to gauge health literacy (ie, “How confident are you filling out medical forms by yourself?” from 1=Extremely to 5=Not at all [19]). Physical and mental health were also assessed. Specifically, history of mental illness was coded as 1 if a participant indicated ever having been diagnosed with depression, schizophrenia or schizoaffective disorder, bipolar disorder, posttraumatic stress disorder, or an anxiety disorder besides posttraumatic stress disorder (all others were coded as 0). Similarly, those who reported ever receiving a diagnosis of a chronic disease, including cardiovascular diseases, pulmonary diseases, cancers, high blood pressure, diabetes, or arthritis, were coded as 1 (all others were coded as 0).

The 4-week follow-up assessment included many items that were assessed at baseline and some additional items. Participants completed the system usability scale (SUS) [20], a 10-item measure that is commonly used to assess the usability of smartphone applications (scores range from 0 to 100, with higher scores indicating greater usability; scores greater than 68 are considered above average, and scores above 80 are in the top 10th percentile) [21]. In addition, participants were asked several questions to assess other qualities of the study smartphone application. Specifically, participants were asked: “Consider the number of assessments that were automatically pushed by the smartphone application, was the number of assessments: 1=Too high, 2=About right, 3=Not enough”; “Did carrying the phone and answering questions make you more aware of your thoughts, feelings, and behavior?” with answer options ranging from 1=Definitely no to 4=Definitely yes; and “Do you find the smartphone application to be annoying?” with answer options ranging from 1=Not at all to 5=Extremely.

#### EMA

The EMA methodology used in this study is similar to what was used in our previous studies and by other researchers [22-31]. Throughout the 28 EMA days, the app prompted participants to complete each EMA by ringing and vibrating for 30 seconds. If the participant did not respond after 5 prompts, the EMA was automatically rescheduled, and participants were prompted again up to 2 additional times 15 minutes later. EMAs asked participants about their mood, environmental context, and health behaviors (eg, fruit and vegetable intake, physical activity, soda consumption). In addition, readiness to change unhealthy behaviors was assessed once per week during the first EMA of the day. Participant GPS locations were collected by the app multiple times per day to be used in future studies that examine location-based effects on mood, thoughts, and behaviors.

#### Additional Derived Variables

Additional variables were derived to determine if they were related to the EMA completion rate. Specifically, a variable indicating whether each EMA was completed on a weekday (=0) or weekend day (=1), time of day the EMA was completed (ie, morning=5 AM-11:59 AM, afternoon=12 PM-4:59 PM, evening=5 PM-8:59 PM, late night=9 PM-4:59 AM), and urban (=0) vs rural (=1) residence (based on address) were calculated and entered into the dataset. We also calculated a time period variable (*time*) corresponding to whether each EMA was completed during the first 14-day period of the study or the second 14-day period of the study.

### App Programming

The Insight app was developed by the mHealth Shared Resource at the University of Oklahoma Health Sciences Center and NCI Designated Stephenson Cancer Center. The Insight mHealth platform enables researchers to rapidly build, test, and launch technology-based assessment and intervention tools. The mHealth resource employs 9.5 staff members, including 5.5 computer scientists and engineers who develop and maintain web and mobile applications and relational databases.

### Ethical Considerations

This study was approved by the University of Oklahoma Health Sciences Center Institutional Review Board (13684). All participants provided informed consent and were informed that they could opt out of the study at any time. Study datasets were deidentified. In addition to compensation for the baseline assessment (US $10 payments made via a reloadable Greenphire Clincard) and follow-up assessment (US $30 via Greenphire Clincard), all participants were compensated at study completion for either the percentage of EMAs that they completed or US $1 per EMA completed over the 4-week study period. Participants in the 4 EMAs per day groups could earn up to $112 for completing EMAs, and those in the 2 EMAs per day groups could earn up to US $56 for completing EMAs. Those randomly assigned to the groups that were paid based upon the percentage of EMAs completed received US $112 (4 per day groups) or US $56 (2 per day groups) for completing 95%-100% of the EMAs, US $90 (4 per day groups) or US $45 (2 per day groups) for completing 80%-94% of the EMAs, US $56 (4 per day groups) or US $28 (2 per day groups) for completing 50%-79% of the EMAs, and $0 for completing less than 50% of the EMAs. Thus, considering compensation for the baseline and follow-up assessments and EMAs, participants could earn up to US $96-$152 for the entire study. Study Greenphire cards were mailed to participants upon completion of the baseline assessment, and compensation was loaded onto the card after the participant indicated that they had received the card. A final follow-up and EMA completion payment were loaded onto the card after the follow-up assessment was completed.

### Statistical Methods

Descriptive statistics were used to summarize participant demographics and engagement with the smartphone app. For all primary analyses, the percentage of EMAs completed over each of the two 14-day study periods was used as the primary outcome variable. Specifically, for each participant during each of the 2 study periods, the number of completed EMAs was divided by the number of total possible EMAs (ie, either 28 or 56, depending on whether the number of EMAs per day was set to 2 or 4) and multiplied by 100. Two compliance scores per person were calculated (instead of 1 total compliance score) to accommodate the within-person nature of the EMA response scale factor.

To examine how each of the design factors related to EMA compliance, we used multilevel (2-level) linear regression, while adjusting for several hypothesized person-level characteristics, including age, Hispanic ethnicity, race, sex, education level, employment status, income, rural versus urban address, current depression, current anxiety, mental diagnosis history, chronic illness diagnosis history, substance use problem history, health literacy, and SUS score. Effect coding was used for each of the 5 main effects: EMA payment type (–1=percentage-based payment, +1=$1 for each EMA), number of EMAs per day (–1=2 EMAs per day, +1=4 EMAs per day), number of items per EMA (–1=15 items, +1=25 items), prompt schedule (–1=random prompts, +1=fixed prompts), and EMA response scale (–1=Likert-type scale, +1=slider-type scale). All design factors, except for the EMA response scale, were at the between-person level in the model (level 2) and only varied between participants. The EMA response scale was at the within-person level in the model (level 1). In the main model, all within-level and cross-level interactions (main effects, 2-way, 3-way, 4-way, and 5-way) were allowed among the design factors. Additionally, time was added as a covariate at the within-person level (level 1; –1=first 14-day period, +1=second 14-day period). An initial adjusted model additionally accounted for between-person variation in EMA compliance due to the person-level characteristics described earlier. A final adjusted model was calculated by using backwards elimination (threshold set to *P*<.10) to identify which of the demographic and environmental predictors from the above list were independently related to EMA completion. A priori power analyses indicated that a sample size of 416 would allow us to detect meaningful differences in EMA completion rates. For example, these analyses indicated 0.81 power to detect a 12-percentage point difference in EMA completion rates of 70% versus 82% or a 10-percentage point difference in EMA completion rates of 80% versus 90%. These base rates were consistent with prior research [13].

To examine whether weekday and time of day were associated with EMA compliance, we created a dichotomous variable to represent whether or not an EMA was prompted on a weekday versus a weekend day, and we categorized EMAs based on the actual notification time on the participant’s phone, with morning defined as 5 AM-11:59 AM, afternoon defined as 12 PM-4:59 PM, evening defined as 5 PM-8:59 PM, and late night defined as 9 PM-4:59 AM. EMAs in which the notification time was missing (eg, due to the phone being off or an app error) were excluded. We used multilevel logistic regression with EMA completion (yes or no) as the outcome, and time of day and weekday versus weekend as the independent variables in 2 separate models.

Finally, to examine whether participant compliance was associated with perceptions of the app (ie, SUS score, answers to whether the number of assessments was too high, whether they felt the app was annoying, and if the app made them more aware of their thoughts, feelings, and behaviors), we used Pearson correlation coefficients and independent samples t-tests. All analyses were performed using R Statistical Software (version 4.1.1; R Foundation for Statistical Computing).

## Results

### Participants

**Figure 3:**
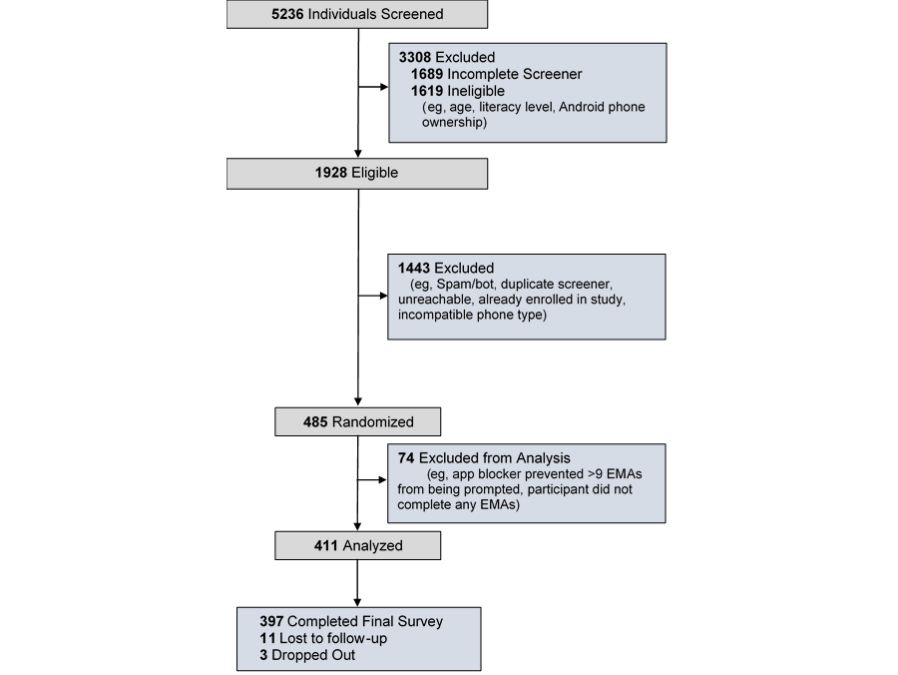
CONSORT (Consolidated Standards of Reporting Trials) table. EMA: ecological momentary assessment.

The study advertisement was clicked 5236 times, and 3547 individuals completed the study screener. A total of 1928 individuals were prescreened eligible to participate in this study, and 485 were enrolled and randomized into 1 of 32 study groups. Since the main purpose of this study was to examine the effects of specific factors on EMA completion rates, the decision was made to not include participants who experienced unusual technical difficulties in which their phones did not prompt 10 or more of the scheduled EMAs (eg, phone specific software, including app blockers, on participant phones prevented app prompts). A total of 69 participants were excluded due to this issue, and an additional 5 were excluded for not completing any EMAs. See Figure 3 for the study CONSORT table.

**Figure 4:**
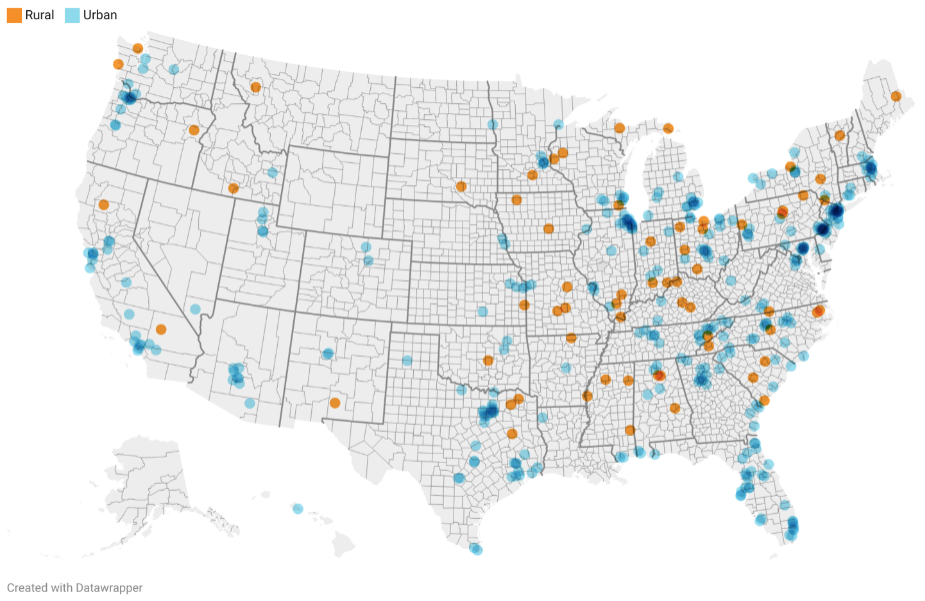
Participant zip codes by rural and urban residence.

Participants (N=411) were mostly female (311/411, 75.7%), White (298/411, 72.5%), and on average 48.4 (SD 12.1) years of age (see Table 2 for participant demographic characteristics). Participants were enrolled from nearly all US states, but most participants were from the East and West coasts (Figure 4). Nearly all participants (397/411, 96.6%) completed the end of study follow-up assessment.

**Table 2:** Demographic characteristics.

			Payment		Number of EMAs^a^/day		Number of items/EMA		EMA schedule	
		Total sample	Percentage-based (–1)	US $1/EMA (+1)	2/day (–1)	4/day (+1)	15 Items (–1)	25 Items (+1)	Random (–1)	Fixed (+1)
Sample size, n		411	206	205	205	206	206	205	206	205
Age (years), mean (SD)		48.4 (12.1)	49.2 (11.9)	47.5 (12.1)	48.2 (12.2)	48.5 (12.0)	48.6 (12.4)	48.2 (11.8)	47.4 (11.7)	49.4 (12.4)
Hispanic/Latino, %		9.6	8.0	11.3	10.8	8.3	7.7	11.4	9.7	9.5
**Race, %**										
	American Indian	1.5	1.5	1.5	1.5	1.5	1.5	1.5	1.5	1.5
	Asian	2.7	1.5	3.9	3.9	1.5	3.9	1.5	1.9	3.4
	Black	18.0	21.8	14.1	15.5	20.5	16.6	19.4	19.4	16.6
	White	72.5	69.4	75.6	73.8	71.2	72.7	72.3	70.4	74.6
	Other/more than 1 race	5.4	5.8	4.9	5.3	5.4	5.4	5.3	6.8	3.9
Female, %		75.7	74.8	76.6	75.7	75.6	76.6	74.8	76.2	75.1
Years of education, mean (SD)		14.5 (2.2)	14.6 (2.2)	14.4 (2.1)	14.6 (2.2)	14.5 (2.1)	14.5 (2.1)	14.5 (2.2)	14.6 (2.1)	14.5 (2.2)
Full- or part-time employed, %		51.8	49.0	50.7	51.9	47.8	49.8	50.0	51.5	48.3
Household income <US $30,000/year, %		29.7	31.1	28.3	29.6	29.8	26.3	33.0	27.2	32.2
Rural residence, %		15.3	15.0	15.6	15.5	15.1	16.6	14.1	15.5	15.1
Probable depression via ODSIS^b^, %		34.1	33.8	34.4	34.5	33.7	34.9	33.3	35.4	32.8
Probable anxiety via OASIS^c^, %		36.6	35.3	42.1	39.9	37.3	38.5	38.8	41.0	36.3
History of mental illness, %		58.6	60.2	57.1	52.9	64.4	58.0	59.2	60.2	57.1
History of chronic illness, %		67.6	73.8	61.5	63.6	71.7	68.8	66.5	66.0	69.3
History of substance use problem, %		14.4	15.0	13.7	12.6	16.1	13.7	15.0	16.0	12.7
Health literacy, mean (SD)		4.8 (0.6)	4.7 (0.6)	4.7 (0.6)	4.8 (0.7)	4.7 (0.5)	4.8 (0.5)	4.7 (0.6)	4.8 (0.5)	4.7 (0.7)
SUS score, mean (SD)		82.7 (13.2)	79.6 (13.4)	85.8 (12.3)	83.2 (12.8)	82.3 (13.6)	82.4 (12.8)	83.1 (13.6)	84.1 (12.7)	81.3 (13.6)

^a^EMA: ecological momentary assessment.

^b^ODSIS: Overall Depression Severity and Impairment Scale.

^c^OASIS: Overall Anxiety Severity and Impairment Scale.

### Effects of Experimental Factors on EMA Completion Rates

Over the 28-day study period, participants completed 83.8% of all scheduled EMAs (28,948 completed EMAs/34,552 total possible EMAs). See Table 3 for EMA completion rates by each study factor. Results indicated no significant main effects or interactions (2-way, 3-way, 4-way, or 5-way), as shown in the columns of Table 4 labeled as unadjusted models. On average, participants earned US $102. The 2 EMAs per day group earned an average of US $83, while the 4 EMAs per day group earned an average of US $130.

**Table 3:** Compliance rates by ecological momentary assessment (EMA) design factor.

Design factor and condition		EMA completion rate, mean (SD)	
**Payment**			
	Percentage-based (–1)		83.0 (18.8)
	$1 Per EMA (+1)		83.9 (13.3)
**Number of EMAs^a^/day**			
	2/day (–1)		82.4 (16.9)
	4/day (+1)		84.5 (15.6)
**Number of items/EMA**			
	15 Items (–1)		84.5 (15.7)
	25 Items (+1)		82.4 (16.9)
**EMA schedule**			
	Random (–1)		83.0 (16.0)
	Fixed (+1)		83.9 (16.6)
**Scale type**			
	Slider		83.8 (16.8)
	Likert		84.3 (15.7)

^a^EMA: ecological momentary assessment.

**Table 4:** Linear multilevel regression models^a^ for compliance rates by EMA^b^ design factors (unadjusted model) and EMA design factors plus demographic characteristics (adjusted model).

			Unadjusted model^c^						Initial adjusted model^d^							Final adjusted model^e^				
			Estimate		SE	*P* value		Estimate			SE		*P* value		Estimate			SE		*P* value
**Fixed**																				
	Intercept	83.7		0.8		<.001	*87.5* ^f^			2.3		<.001		*87.4*			0.8		<.001	
	Paid per EMA (+1)	0.1		0.8		.80	–*1.3*			0.7		.04		–1.3			0.6		.10	
	4 EMAs per day (+1)	0.9		0.8		.20	0.4			0.6		.60		0.2			0.6		.70	
	25 Items per EMA (+1)	–1		0.8		.20	–1.2			0.6		.10		–1.1			0.6		.10	
	Fixed schedule (+1)	0.5		0.8		.50	0.3			0.6		.70		0.2			0.6		.70	
	Slider-type response	0.4		0.3		.20	0.3			0.3		.40		0.3			0.3		.40	
	Time	–0.6		0.3		.10	–0.4			0.3		.20		–0.4			0.3		.20	
	Paid per EMA×4 EMAs per Day	0.4		0.8		.60	–0.1			0.6		.90		–0.1			0.6		.90	
	Paid per EMA×25 items per EMA	0.3		0.8		.70	0.3			0.6		.60		0.4			0.6		.50	
	Paid per EMA×fixed schedule	0.9		0.8		.20	0.4			0.6		.50		0.6			0.6		.40	
	4 EMAs per day×25 items per EMA	0.1		0.8		.90	0.2			0.6		.70		0.1			0.6		.90	
	4 EMAs per day×fixed	–0.1		0.8		.90	0.1			0.6		.90		–0.1			0.6		.90	
	25 Items per EMA×fixed schedule	–0.8		0.8		.30	0			0.6		>.99		–0.2			0.6		.80	
	Slider-type response×paid per EMA	–0.1		0.3		.80	–0.1			0.3		.80		–0.1			0.3		.80	
	Slider-type response×4 EMAs per day	–0.2		0.3		.60	–0.1			0.3		.70		–0.1			0.3		.70	
	Slider-type response×25 Items per EMA	–0.4		0.3		.30	–0.5			0.3		.10		–0.5			0.3		.10	
	Slider-type response×fixed schedule	0.1		0.3		.70	0.2			0.3		.60		0.2			0.3		.60	
	Age	—		—		—	0.1			0.1		.10		*0.1*			0.1		.02	
	Hispanic or Latino	—		—		—	–2.5			2.2		.30		—			—		—	
	White	—		—		—	1			1.5		.50		—			—		—	
	Female	—		—		—	1.2			1.6		.50		—			—		—	
	Years of education	—		—		—	0.6			0.3		.10		—			—		—	
	Full- or part-time employed	—		—		—	–2.6			1.5		.10		—			—		—	
	Household income <US $30,000/year	—		—		—	–1.6			1.7		.30		—			—		—	
	Rural residence	—		—		—	0.7			1.8		.70		—			—		—	
	Probable depression via ODSIS^g^	—		—		—	–*4.4*			1.8		.01		–*3.9*			1.3		.004	
	Probable anxiety via OASIS^h^	—		—		—	0.1			1.8		>.99		—			—		—	
	History of mental illness	—		—		—	1.4			1.6		.40		—			—		—	
	History of chronic illness	—		—		—	–0.8			1.6		.60		—			—		—	
	History of substance use problem	—		—		—	–3.2			1.9		.10		–*3.9*			1.8		.03	
	Health literacy	—		—		—	1.0			1.2		.40		—			—		—	
	SUS^i^ score	—		—		—	*0.2*			0.1		<.001		*0.2*			0.1		<.001	

^a^Models included all main effects and interactions (ie, 2-way, 3-way, 4-way, and 5-way).

^b^EMA: ecological momentary assessment.

^c^Random intercept SD estimate 13.5 (95% CI 12.2-14.8); random residual SD estimate 9.9 (95% CI 9.2-10.6).

^d^Random intercept SD estimate 10.5 (95% CI 9.5-11.7); random residual SD estimate 8.7 (95% CI 8.1-9.4).

^e^Random intercept SD estimate 10.6 (95% CI 9.6-11.7); random residual SD estimate 8.7 (95% CI 8.1-9.4).

^f^All significant results are italicized.

^g^ODSIS: Overall Depression Severity and Impairment Scale.

^h^OASIS: Overall Anxiety Severity and Impairment Scale.

^i^SUS: system usability scale.

### Relations Among Demographics, Environmental Variables, and EMA Completion Rates

#### Overview

An initial adjusted linear multilevel regression model was fit to examine which demographic factors were associated with EMA compliance, after accounting for EMA design factors (see Table 4, Initial adjusted model). After backwards elimination was used to identify which demographic factors had the largest association with EMA compliance (Table 4, Final adjusted model), results indicated that age was positively associated with EMA compliance, such that for every year increase in age, EMA compliance was expected to increase by 0.1% (*P*=.02). Both current depression via the Overall Depression Severity and Impairment scale (β=–3.9, *P*=.004) and substance use problem history (β=–3.9, *P*=.03) were associated with lower EMA compliance. Finally, the app usability score was positively associated with EMA compliance (β=.2, *P*<.001).

#### EMA Compliance by Day of Week

On average, participants completed 84.5% (20,807/24,625) of EMAs that were prompted on weekdays and 82.6% (8137/9852) of EMAs prompted on weekend days. Results showed that the log odds of completing EMAs prompted on weekdays were significantly higher than the log odds of completing an EMA prompted on a weekend (β=.16, 95% CI 0.09-0.23, *P*<.001).

#### EMA Compliance by Time of Day

Of the prompts that occurred in the morning, 87.3% (10,183/11,669) were completed. Of the prompts that occurred in the afternoon, 88.8% (6415/7227) were completed. Of the prompts that occurred in the evening, 89.9% (6781/7544) were completed. Of the prompts that occurred in the late night, 88.5% (5567/6292) were completed. Results from fitting a multilevel logistic model indicated that the log odds of completing an EMA prompted in the morning (the reference group) were 2.2 (*P*<.001; 95% CI 2.1-2.3). Further, the results showed that the log odds of completing EMAs prompted in the afternoon (β=.2, 95% CI 0.1-0.3, *P*<.001), evening (β=.3, 95% CI 0.2-0.4, *P*<.001), and late night (β=.2, 95% CI 0.1-0.3, *P*<.001) were significantly higher than the log odds of completing an EMA prompted in the morning. Refitting the model with afternoon as the reference group indicated that the log odds of completing EMAs prompted in the evening were also significantly higher than the log odds of completing EMAs prompted in the afternoon (β=.114, 95% CI 0.002-0.227, *P*=.046). In contrast, EMAs prompted in the late night were not statistically different from those prompted in the afternoon (β=–.04, 95% CI –0.16 to 0.08, *P*=.55). Refitting the model once more with evening as the reference group showed that the log odds of completing EMAs prompted in the late night were significantly lower than those prompted in the evening (β=–.15, 95% CI –0.27 to 0.03, *P*=.01).

### Participant Perceptions of EMAs

Overall, the Insight app was well liked, as measured by the SUS (mean 82.7, SD 13.2), and the SUS score was positively related to the overall EMA completion rate (*r*=0.14, *P*=.005). A minority of participants (71/397, 17.9%) reported that the number of EMAs that they were prompted to complete was too high. Those assigned 4 EMAs per day (*P*=.001), paid by percentage of EMAs completed (*P*<.001), and on a fixed EMA schedule (*P*=.03) were more likely to indicate that the number of EMAs was too high. A minority of participants (86/397, 21.7%) indicated that the app was moderately to extremely annoying. Those assigned to receive payment by percentage of EMAs completed were more likely to find the smartphone application annoying (*P*=.002) compared to those who were paid US $1 per EMA completed. Most participants (336/397, 84.6%) reported that answering EMA questions made them more aware of their thoughts, feelings, and behavior. Those assigned to receive payment per EMA completed were more likely to indicate that answering EMA questions made them more aware of their thoughts, feelings, and behavior (*P*=.03) compared with those who were paid US $1 per EMA completed.

### App Engagement

On average, participants clicked the payment button 66.7 (SD 61.8) times and the app instructions button 9.2 (SD 11.3) times. The average number of Payment button clicks was much higher for those assigned to be paid based upon the percentage of EMAs completed (mean 77.2) than those assigned to receive US $1 per EMA (mean 56.1; *t*=–3.509, *P*<.001).

## Discussion

### Principal Findings

This study examined the impact of EMA design features on participant compliance in a prospective, experimental design. The results of this study demonstrated that EMA frequency (2 vs 4 EMAs per day), number of EMA items (15 vs 25 questions), EMA prompt type (fixed vs random), payment type (US $1 per EMA vs payment by percentage of EMAs completed), and EMA response type (slider-type responses vs Likert-type responses) were not associated with EMA completion rates. Prior research on the impact of EMA design factors on compliance has been mixed. For example, Jones et al [8] similarly found that EMA compliance was not associated with prompt frequency, while 2 other meta-analyses found that fewer prompts were associated with increased compliance rates [14,15]. Further, while Vachon et al [14] found that fixed EMA prompts were associated with higher rates of compliance, our results suggest that there was no significant difference in compliance between fixed versus random prompt types. These differences may indicate that there are other confounding factors and individual variability that impact EMA compliance beyond design factors such as frequency or length of assessments, such as variations in participants’ daily schedules, or changes in motivation, attention span, or engagement in the study.

We also examined the impact of several demographic and environmental characteristics on the EMA completion rate, and used backwards elimination to identify the factors that had the largest association with EMA completion. Age, history of a substance use problem, probable current depressive disorder, and SUS score were the factors that were most associated with EMA completion, accounting for experimental factors. While prior research has suggested that older adults may experience barriers related to smartphone use such as lack of interest and technological knowledge [32,33], our results suggested that older age was associated with higher levels of EMA completion. Our results are similar to those of Ono et al [34] and Cain et al [35], which found that EMA protocols are generally feasible and acceptable to older adults. Further, we found that adults without a history of a substance use problem completed 5.9% more EMAs on average than those with a history of substance use problems (ie, 84.3% vs 78.4%). This is consistent with a 2019 meta-analysis of compliance with EMA protocols among substance users, which found that substance-dependent samples had lower compliance rates compared to nondependent samples [8]. In addition, individuals with probable current depression had lower EMA completion rates than those without current depression. Although this effect was statistically significant, current probable depression was related to a 2.1% average difference (84.3% vs 82.2%) in EMA completion over the 28-day study period and thus should not be overstated. There were no differences in EMA completion by race, ethnicity, current anxiety, rural versus urban residency, income, years of education, history of chronic illness, history of mental illness, or health literacy. These findings suggest that EMA protocols may be feasible among a diverse group of individuals and that limitations that may impact participation in traditional health behavior interventions, such as living in a rural area or lower health literacy, may not be barriers to smartphone-based studies. However, for studies that use EMA to inform the delivery of health behavior interventions (eg, JITAIs), it may be beneficial to plan EMA prompts based on an individual’s actual availability (eg, outside of work hours) to maximize engagement with EMA protocols.

There were no main effects of EMA frequency, number of EMA items, EMA prompt type, payment type, or EMA response scale type on EMA completion rates. These study results and several others have implications for future EMA study designs. First, while payment type did not impact EMA completion rates, participants assigned to receive compensation based upon the percentage of EMAs completed tended to engage with the app “Payment” button more frequently than those assigned to receive $1 per EMA. This may indicate that participants assigned to the percentage condition frequently clicked the payment button to determine if they had missed any EMAs. For studies in which engagement with an app is important (eg, to view intervention content), a payment based on the percentage of EMAs completed may increase time spent in the app or button clicks. In addition, we found that participant perceptions of the Insight app were extremely positive via the SUS. In fact, SUS scores were in the top 10th percentile [21] and SUS scores were positively associated with EMA completion. Therefore, an individual’s confidence in using a specific app, its ease of use, and its perceived usability may be important factors for EMA completion rates. Finally, our results demonstrated that EMA compliance was higher on weekdays compared to weekends and that there were differences in compliance by time of day, with the highest level of compliance for evening assessments compared with morning, afternoon, and late-night assessments. This finding suggests that participants may be more engaged during the week, and that evening assessments may be more amenable to participants. Upon study setup, participants were able to specify sleep and wake times for each day of the week so that they would only receive EMAs during waking hours. This type of customization may be particularly important for shift workers, and individuals who work night shifts.

### Study Strengths and Limitations

The strengths of this study include its prospective, randomized design and large sample size drawn from the entire United States (ie, participants were recruited from all but 4 states). However, there are several limitations. First, our sample was primarily non-Hispanic White and female, so our findings may not be generalizable to other populations. It should be noted, however, that we did not find any significant difference in EMA completion rates by sex or race. Second, the Insight app, at the time of data collection, was only compatible with phones using the Android operating system. However, it is important to keep in mind that, as of 2022, Android accounted for 71.8% of the worldwide mobile operating system market share [36]. An additional limitation is that individuals who sign up for remote smartphone-based studies and individuals recruited via Facebook may be different from the general population. Specifically, individuals who are more comfortable with technology, have a Facebook account, and do not perceive that smartphone-based surveys are burdensome may be more likely to sign up for an EMA study than individuals who are not comfortable with smartphones. Findings indicated that there was a very high proportion of participants with current anxiety or depression. This finding is consistent with the significant increase in anxiety and depression that was reported during the COVID-19 pandemic [37,38]. Finally, it was unexpected that all of the factors that were examined were not related to EMA compliance. These findings may indicate that the differences between each level of these design factors (eg, 15 vs 25 questions) were not large enough to result in significant differential completion rates of prompted EMAs based on the study sample size. Bigger differences in factors like the number of EMAs within a day [14,15] or the number of questions per EMA [15] could result in greater differentiation in EMA compliance. However, the factors, levels of each factor, and duration of the EMA period (ie, 28 days) were specifically selected and tuned to assess compliance with and acceptability of EMA schedules that could be embedded into future smartphone-based JITAI that use EMA data to tailor intervention content.

### Future Work

Future analyses from these data will evaluate whether slider-type and Likert-type response scales can be interchangeably used to predict daily health behaviors, whether there are differences in the predictive value of data collected during fixed and random EMAs, and if there is additional value in asking participants to complete 4 versus 2 EMAs per day.

### Conclusions

Participants were highly compliant with a 28-day EMA design that varied across 5 EMA design dimensions. Studies that incorporate EMA data collection methods must balance the benefits of obtaining granular, real-time data about participants’ thoughts, feelings, and behaviors with the participant burden of an intensive survey protocol. The results of this study indicated that all of the factors that were examined (ie, number of EMA items, EMA frequency, EMA payment type, EMA prompt type, and EMA response scale type) did not significantly impact EMA completion rates, nor did multiple demographic characteristics (eg, race or ethnicity or urban vs rural), history of mental illness, or history of other chronic illnesses. However, younger adults, those with current depression, and those with a history of a substance use problem completed fewer EMAs than their counterparts. EMA studies may benefit from customizing protocols to the behaviors, phenomena, or populations being studied, without arbitrary limitations on design factors. For example, the frequency and timing of EMA prompts should match the anticipated frequency and diversity of the behavior being assessed.

## Appendix

Multimedia Appendix 1 Ecological momentary assessment conditions.

## Abbreviations

EMA: ecological momentary assessment
JITAI: just-in-time adaptive intervention
REDCap: Research Electronic Data Capture
SUS: system usability scale

## Data Availability

Data from the Exemplar study are not publicly available. Deidentified data are available upon reasonable requests made to the lead author of this paper.
