# Supplementary material for: Investigating Best Practices for Ecological Momentary Assessment: Nationwide Factorial Experiment
Source: J Med Internet Res. 2024 Aug 12;26:e50275. doi: 10.2196/50275 (PMC11347889; doi:10.2196/50275)
Supplement: Multimedia Appendix 1 [file jmir_v26i1e50275_app1.docx]

| Multimedia Appendix 1: EMA conditions. | | | | |
| --- | --- | --- | --- | --- |
| **Payment** | **EMAs per day** | **EMA items** | **Schedule** | **Order** |
| 1. $1 Payment per EMA | 2 EMAs per day | 15 Questions per EMA | Fixed schedule | Slider first |
| 1. $1 Payment per EMA | 2 EMAs per day | 15 Questions per EMA | Fixed schedule | Likert first |
| 1. $1 Payment per EMA | 2 EMAs per day | 15 Questions per EMA | Random schedule | Slider first |
| 1. $1 Payment per EMA | 2 EMAs per day | 15 Questions per EMA | Random schedule | Likert first |
| 1. $1 Payment per EMA | 2 EMAs per day | 25 Questions per EMA | Fixed schedule | Slider first |
| 1. $1 Payment per EMA | 2 EMAs per day | 25 Questions per EMA | Fixed schedule | Likert first |
| 1. $1 Payment per EMA | 2 EMAs per day | 25 Questions per EMA | Random schedule | Slider first |
| 1. $1 Payment per EMA | 2 EMAs per day | 25 Questions per EMA | Random schedule | Likert first |
| 1. $1 Payment per EMA | 4 EMAs per day | 15 Questions per EMA | Fixed schedule | Slider first |
| 1. $1 Payment per EMA | 4 EMAs per day | 15 Questions per EMA | Fixed schedule | Likert first |
| 1. $1 Payment per EMA | 4 EMAs per day | 15 Questions per EMA | Random schedule | Slider first |
| 1. $1 Payment per EMA | 4 EMAs per day | 15 Questions per EMA | Random schedule | Likert first |
| 1. $1 Payment per EMA | 4 EMAs per day | 25 Questions per EMA | Fixed schedule | Slider first |
| 1. $1 Payment per EMA | 4 EMAs per day | 25 Questions per EMA | Fixed schedule | Likert first |
| 1. $1 Payment per EMA | 4 EMAs per day | 25 Questions per EMA | Random schedule | Slider first |
| 1. $1 Payment per EMA | 4 EMAs per day | 25 Questions per EMA | Random schedule | Likert first |
| 1. Payment by % EMAs | 2 EMAs per day | 15 Questions per EMA | Fixed schedule | Slider first |
| 1. Payment by % EMAs | 2 EMAs per day | 15 Questions per EMA | Fixed schedule | Likert first |
| 1. Payment by % EMAs | 2 EMAs per day | 15 Questions per EMA | Random schedule | Slider first |
| 1. Payment by % EMAs | 2 EMAs per day | 15 Questions per EMA | Random schedule | Likert first |
| 1. Payment by % EMAs | 2 EMAs per day | 25 Questions per EMA | Fixed schedule | Slider first |
| 1. Payment by % EMAs | 2 EMAs per day | 25 Questions per EMA | Fixed schedule | Likert first |
| 1. Payment by % EMAs | 2 EMAs per day | 25 Questions per EMA | Random schedule | Slider first |
| 1. Payment by % EMAs | 2 EMAs per day | 25 Questions per EMA | Random schedule | Likert first |
| 1. Payment by % EMAs | 4 EMAs per day | 15 Questions per EMA | Fixed schedule | Slider first |
| 1. Payment by % EMAs | 4 EMAs per day | 15 Questions per EMA | Fixed schedule | Likert first |
| 1. Payment by % EMAs | 4 EMAs per day | 15 Questions per EMA | Random schedule | Slider first |
| 1. Payment by % EMAs | 4 EMAs per day | 15 Questions per EMA | Random schedule | Likert first |
| 1. Payment by % EMAs | 4 EMAs per day | 25 Questions per EMA | Fixed schedule | Slider first |
| 1. Payment by % EMAs | 4 EMAs per day | 25 Questions per EMA | Fixed schedule | Likert first |
| 1. Payment by % EMAs | 4 EMAs per day | 25 Questions per EMA | Random schedule | Slider first |
| 1. Payment by % EMAs | 4 EMAs per day | 25 Questions per EMA | Random schedule | Likert first |
